# Olfactory Training for Post-COVID-19 Olfactory Dysfunction: A Meta-Analysis of Efficacy and Combination Therapies

**DOI:** 10.3390/jcm14186578

**Published:** 2025-09-18

**Authors:** Ali Alsuheel Asseri, Mona Aldukain, Ali Aldukain, Abdulmohsin Alzuhairi

**Affiliations:** 1 Department of Child Health, College of Medicine, King Khalid University, Abha 62521, Saudi Arabia; 2 Faculty of Medicine, King Khaled University, Abha 62529, Saudi Arabia; mona.aldukain11@gmail.com (M.A.); dr.alihq@gmail.com (A.A.); hsoon2277@gmail.com (A.A.)

**Keywords:** olfactory training, post-COVID-19, olfactory dysfunction, meta-analysis, combination therapies

## Abstract

**Background/Objectives**: This systematic review and meta-analysis evaluated the effectiveness of olfactory training (OT) using standardized protocols in patients with post-COVID-19 olfactory dysfunction. The objective was to assess whether OT, compared to no treatment, placebo, or alternative therapies, improved olfactory function as measured using validated smell tests, including UPSIT, Sniffin’ Sticks (TDI score), CCCRC, and B-SIT. **Methods**: A systematic search of PubMed, Web of Science, and Ovid Medline was conducted through February 2025 in accordance with PRISMA guidelines. Eight randomized controlled trials (RCTs) met the inclusion criteria. Data were extracted on study characteristics (author, year, country, design, sample size), population details (age, sex, post-COVID-19 cause), intervention type (training method, frequency, duration), comparators, outcome measures (baseline and post-intervention olfactory scores), follow-up duration, and reported adverse effects. The risk of bias was assessed using the Joanna Briggs Institute critical appraisal tool. Meta-analyses were performed using RevMan and Open Meta-Analyst. **Results**: Olfactory training significantly improved the olfactory scores compared to those of the controls. The greatest improvement was observed when OT was combined with PEA-luteolin (MD = 4.62, 95% CI: 2.17–7.06, *p* = 0.0002), followed by EDTA (MD = 2.33, 95% CI: 0.58–4.08, *p* = 0.009). Corticosteroids showed a borderline benefit (MD = 1.34, 95% CI: 0.01–2.67, *p* = 0.05), while alpha-lipoic acid had no significant effect. Combination therapies were associated with higher recovery rates (RR = 1.65, 95% CI: 1.13–2.42, *p* = 0.01). **Conclusions**: Olfactory training is an effective treatment for post-COVID-19 smell dysfunction. When paired with specific adjunct therapies, particularly PEA-luteolin, it may yield superior recovery outcomes. Further large-scale, standardized RCTs are needed to define optimal treatment protocols.

## 1. Introduction

Olfactory dysfunction is a prevalent and often prolonged symptom of COVID-19, which affects a considerable number of patients [1,2]. As a significant component of the considerable burden of post-acute sequelae of COVID-19 (PASC), early epidemiological surveys indicate that a substantial proportion of infected individuals experience anosmia or hyposmia [3]. The authors studied 751 patients (477 females, 274 males; mean age 41 ± 13 years); a substantial proportion reported a subjective loss of smell: 83% (*n* = 621) experienced total anosmia, and 17% (*n* = 130) reported partial hyposmia. Following a mean follow-up of 47 ± 7 days, olfactory function had completely recovered in 49% (*n* = 367) of patients, while 14% (*n* = 107) reported partial recovery, and 37% (*n* = 277) experienced persistent dysfunction. The mean duration of olfactory dysfunction for those with complete recovery was 10 ± 6 days, compared to 12 ± 8 days for those with partial recovery [3]. These findings confirm that olfactory dysfunction is a frequent and often prolonged sequela of COVID-19 infection [3]. Additionally, while spontaneous recovery occurs in many, a significant subset develops chronic severe hyposmia or functional anosmia that persists long after the acute phase. This protracted sensory impairment has been shown to correlate with a marked reduction in both general health-related and olfaction-specific quality of life, impacting daily activities and contributing to mental health issues such as depression and anxiety, as evidenced by numerous studies [4,5]. Given these challenges, effective treatments are urgently required.

Olfactory training has emerged as a promising non-pharmacological intervention for post-viral smell loss, including COVID-19-related dysfunction [6]. It involves repeated exposure to certain odors over time in order to stimulate neural plasticity and aid recovery [7] and has shown potential benefits for patients with persistent olfactory dysfunction following COVID-19 [8,9]. Research suggests that structured exposure to different odors may enhance regeneration and improve olfactory function, making it a widely recommended approach [10].

Although numerous therapies are commonly used, pharmacological options such as corticosteroids, nasal irrigation, and other medications have not shown significant effectiveness in regaining patients’ sense of smell post-COVID-19 [11,12]. Some research suggested minor benefits from systemic or topical steroids, but their efficacy remains inconsistent, and concerns over side effects restrict their application [13,14].

Although previous reviews have investigated interventions for post-COVID-19 olfactory dysfunction, many have been narrative reviews or included heterogeneous study designs, making it difficult to draw definitive conclusions. Additionally, the effectiveness of olfactory training has not been comprehensively evaluated through a meta-analysis focused on randomized controlled trials (RCTs). Considering these gaps, a systematic review and meta-analysis are required in order to evaluate the actual effectiveness of olfactory training compared to control or other interventions in post-COVID-19 smell dysfunction. Such an analysis should focus on studies that assess quantitative olfactory disorders using validated scoring systems for the primary endpoint, in order to minimize subjectivity associated with reporting qualitative symptoms. By synthesizing evidence from randomized controlled trials, this review will offer evidence-based guidelines for clinicians treating ongoing post-COVID-19 smell loss and enhancing patient outcomes.

## 2. Methods

We performed our systematic review and meta-analysis according to the Preferred Reporting Items for Systematic Reviews and Meta-Analysis (PRISMA) guidelines (version 5.1.0) [15]. The study protocol was pre-registered in the International Prospective Register of Systematic Reviews (PROSPERO) (ID: CRD420251010931).

### 2.1. Literature Search and Data Collection

We conducted our study using three electronic databases: PubMed, Web of Science, and Ovid Medline. We used the following search strategy: (“olfactory training” OR “olfactory rehabilitation”) AND (“anosmia” OR “olfactory dysfunction” OR “smell loss”) AND (“post-COVID-19” OR “COVID-19”) AND (“randomized controlled trial” OR “clinical trial”). Duplications were removed using the Rayyan software (version 1.4.3).

### 2.2. Eligibility Criteria and Study Selection

Two independent reviewers screened the articles by title and abstract, followed by a full-text review, to identify studies that met the inclusion criteria. Eligible studies were randomized controlled trials (RCTs) that investigated adults and pediatric patients with post-COVID-19 olfactory dysfunction. The intervention had to involve standardized olfactory training methods, such as Sniffin’ Sticks (Hummel protocol) or essential oil-based training (Hummel modified protocol), with comparators including placebo, no treatment, or alternative interventions. The primary outcome was olfactory function improvement, assessed using standardized tests like UPSIT, Sniffin’ Sticks (TDI score), CCCRC, and B-SIT. Studies were excluded if they were cohort studies, cross-sectional studies, case reports, reviews, expert opinions, or animal studies, or if they included patients with olfactory dysfunction due to trauma, congenital causes, or neurodegenerative diseases (e.g., Parkinson’s, Alzheimer’s). Additionally, studies using non-standardized or poorly defined training protocols, lacking a comparator group, or assessing olfactory recovery based solely on subjective reporting without standardized testing were not included.

### 2.3. Methodological Quality Assessment

The methodological quality of the included studies was assessed using the JBI Critical Appraisal Tool. Each study was independently evaluated by two reviewers for potential biases, participant selection, and methodology. Any discrepancies were resolved through consensus between the reviewers.

### 2.4. Data Extraction

Two independent reviewers extracted the following data: study details (author, year, country, design, sample size), population characteristics (number of participants, age, sex), intervention (olfactory training method, frequency, duration), comparators, outcome measures (olfactory scores pre- and post-intervention), follow-up duration, and reported adverse effects.

### 2.5. Statistical Analysis

The data analysis was conducted using RevMan (Version 5.3) and Open Meta-Analyst for Microsoft Windows. Our primary effect size measures were mean difference (MD) and risk ratio (RR) with a 95% confidence interval (CI); MD was used to measure changes in olfactory function, while recovery rates were assessed using RR. A *p*-value < 0.05 indicated a statistically significant effect size. To assess heterogeneity, the I^2^ statistic was used. An I^2^ value of 50% with a *p*-value < 0.1 was considered indicative of statistically significant heterogeneity.

## 3. Results

### 3.1. Characteristics of Included Studies

**Figure 1:**
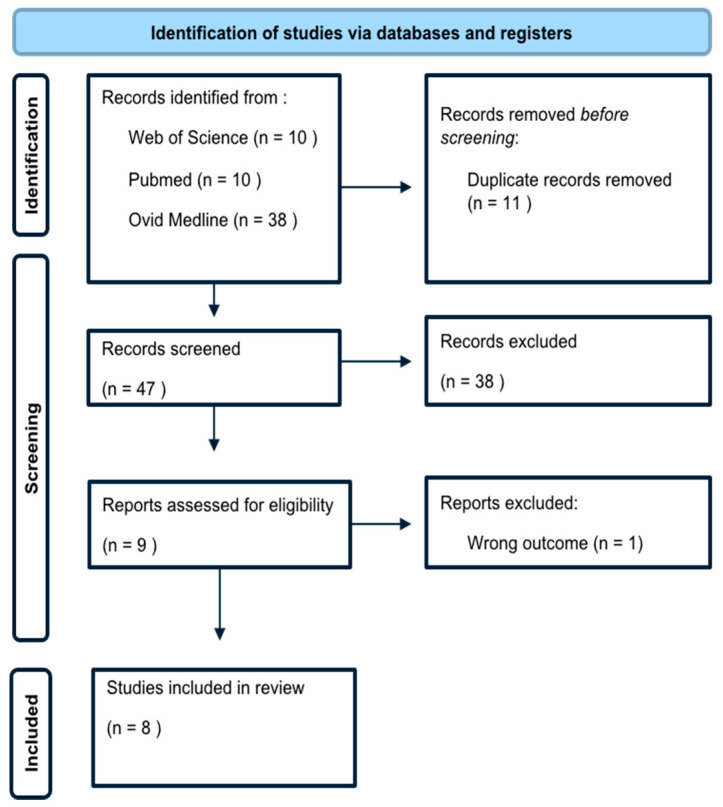
Search and selection process.

A total of 58 studies were initially identified: 10 from PubMed, 38 from Ovid Medline, and 10 from Web of Science. After screening titles and abstracts, nine studies were chosen for full-text review, of which eight fulfilled all the inclusion criteria (Figure 1) [16,17,18,19,20,21,22,23].

Several studies from Egypt, Italy, and Brazil have assessed the efficacy of olfactory training (OT) alone or in combination with adjunct therapies in improving olfactory dysfunction. Training typically involved twice- or thrice-daily exposure to odors such as lemon, rose, clove, and eucalyptus over 3–12 weeks. The interventions ranged from OT alone to combinations with ultramicronized palmitoylethanolamide and luteolin (PEA-LUT), alpha-lipoic acid (ALA), or EDTA nasal spray. Table S1 summarizes the characteristics of the included studies.

### 3.2. Quality Assessment

A quality assessment of the included studies was conducted using the JBI (Joanna Briggs Institute) critical appraisal tool for RCTs. Each study was evaluated across key methodological domains, including randomization, allocation concealment, blinding, outcome measurement, and completeness of follow-up. Most studies demonstrated a low risk of bias, particularly in randomization and outcome assessment, though a few lacked clarity in blinding procedures. Overall, the quality of the included studies ranged from acceptable to high, as summarized in the accompanying quality assessment shown in Table S2.

### 3.3. Change in Olfactory Function

**Figure 2:**
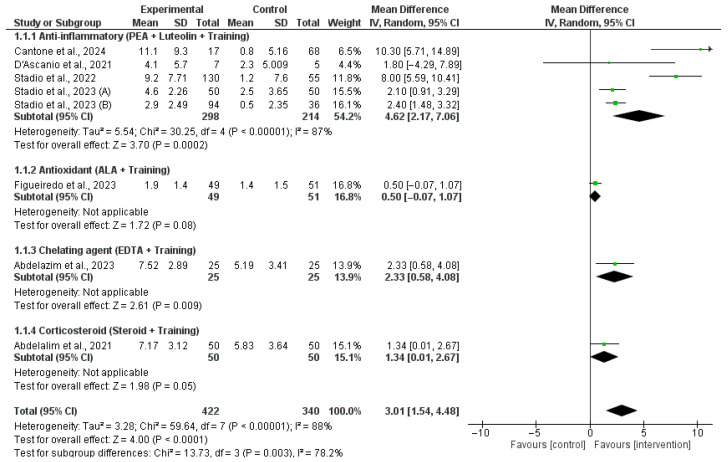
Pooled mean differences in post-intervention olfactory scores comparing olfactory training alone and in combination with adjunct therapies in post-COVID-19 olfactory dysfunction [16,17,18,19,20,21,22,23].

When measuring changes in olfactory function as a continuous outcome (Figure 2), our pooled analysis of mean differences in post-intervention olfactory scores further supported the efficacy of certain combination therapies. The most pronounced improvement was seen with PEA and luteolin combined with olfactory training, which significantly increased olfactory scores compared to controls (MD = 4.62, 95% CI: 2.17–7.06, *p* = 0.0002) despite a high level of heterogeneity (I^2^ = 87%). The addition of EDTA to training also led to a significant improvement in olfactory function (MD = 2.33, 95% CI: 0.58–4.08, *p* = 0.009). While the use of corticosteroids showed a borderline significant benefit (MD = 1.34, 95% CI: 0.01–2.67, *p* = 0.05), ALA supplementation did not significantly enhance olfactory recovery (MD = 0.50, 95% CI: −0.07–1.07, *p* = 0.08). Overall, the combined mean difference for all interventions was statistically significant at 3.01 (95% CI: 1.54–4.48, *p* < 0.0001).

**Figure 3:**
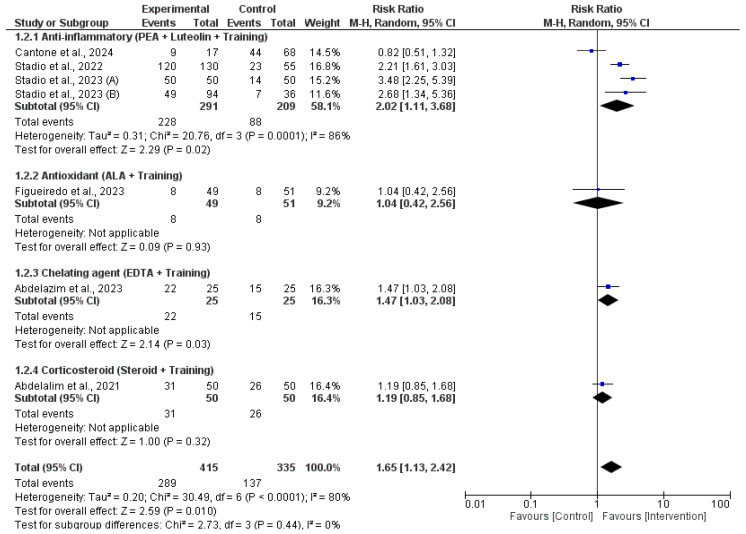
Pooled risk ratios for recovery from post-COVID-19 olfactory dysfunction with olfactory training alone versus in combination with adjunct therapies [16,17,18,19,20,21,22,23].

Our meta-analysis of recovery rates (Figure 3) demonstrated that several adjunct therapies combined with olfactory training significantly improved the likelihood of recovery from olfactory dysfunction when compared to training alone. Among these, anti-inflammatory interventions using ultramicronized palmitoylethanolamide (PEA) and luteolin in combination with olfactory training showed statistically significant benefits, with a pooled RR of 2.02 (95% CI: 1.11–3.68, *p* = 0.02), though heterogeneity was substantial (I^2^ = 86%). Similarly, the use of a chelating agent (EDTA) with training was also effective, yielding an RR of 1.47 (95% CI: 1.03–2.08, *p* = 0.03). In contrast, antioxidant therapy with alpha-lipoic acid (ALA) did not demonstrate a significant benefit (RR = 1.04, 95% CI: 0.42–2.56, *p* = 0.93), nor did the combination of corticosteroids with olfactory training (RR = 1.19, 95% CI: 0.85–1.68, *p* = 0.32). The overall pooled effect across all studies favored the intervention group, with a statistically significant combined RR of 1.65 (95% CI: 1.13–2.42, *p* = 0.01), indicating a 65% greater chance of recovery in patients receiving additional interventions beyond olfactory training alone.

## 4. Discussion

Several studies have documented the significant impact of post-viral olfactory dysfunction on quality of life, with this burden being particularly pronounced in the post-COVID-19 infection [2]. The precise mechanism of post-viral olfactory dysfunction remains an active area of investigation. While the underlying mechanism is not yet fully understood, several theories have proposed a dual pathway for the insult. One hypothesis suggests a direct involvement of the olfactory neuroepithelium, whereby the virus compromises the integrity and function of the supporting cells, thereby disrupting the ability of olfactory sensory neurons to transmit signals. An alternative or complementary theory points to indirect damage mediated by neuroinflammation. This model posits that an inflammatory response within the central nervous system, particularly affecting the olfactory bulb, could lead to a disruption of the olfactory neural circuitry and subsequent persistent dysfunction. Emerging evidence, particularly from post-mortem studies of COVID-19 patients, suggests that a combination of these mechanisms may contribute to the wide spectrum of olfactory symptoms observed [2].

With this systematic review and meta-analysis, we aimed to assess the effectiveness of olfactory training alone or as an adjunctive therapy for post-COVID-19 olfactory dysfunction. Our review gathered data from randomized controlled trials (RCTs) and examined the influence of all interventions on olfactory recovery in patients with anosmia or hyposmia after COVID-19 infection. Our analysis indicates that olfactory training alone or in combination with adjunctive therapies provides a significant benefit to patients with post-COVID-19 smell loss. Specifically, adjunctive therapies including ultramicronized palmitoylethanolamide (PEA) and luteolin showed significant improvements in olfactory scores (mean difference = 4.62, 95% CI: 2.17–7.06, *p* = 0.0002). This suggests that adding an olfactory training program could further improve the outcome of olfactory function recovery through the addition of anti-inflammation therapies, which in turn could enhance neural regeneration and the recovery time for olfactory function [17,23].

Furthermore, when adding corticosteroids to an olfactory training program, there was less of a clinically significant benefit, and the final result showed borderline statistical significance (mean difference = 1.34, 95% CI: 0.01–2.67, *p* = 0.05). This is consistent with the literature, as the value of corticosteroids is often debated since side effects may limit their use in potential long-term treatment regimens for olfactory dysfunction [16,18]. In addition, the meta-analysis of recovery rates showed 65% increased odds of recovery for patients who received olfactory training with adjunct therapies as compared to those patients receiving training alone (RR = 1.65, 95% CI: 1.13–2.42, *p* = 0.01). This adds to the premise that adjunct therapy can enhance olfactory training and that a multimodal therapy may be best for the treatment of post-viral smell dysfunction [19]. The findings of this review are congruent with previous studies, which suggested that olfactory training is a viable treatment option for improving olfactory function in patients with viral infections, including COVID-19 [21,22]. However, although prior studies also suggest multiple adjunct therapies, including PEA, luteolin, and corticosteroids, we have conducted a further pooled analysis of all of these adjunct therapies, specifically noting the importance of combination therapies in improving recovery rates. For instance, it has been suggested and demonstrated that utilizing PEA and luteolin along with olfactory training has provided significant improvements in olfactory function in multiple studies [9,24,25], and our results add further credence to this as well. Conversely, no significant effect of alpha-lipoic acid (ALA) supplementation was noted, consistent with the previous literature questioning the role of this therapy in the recovery of the sense of smell post-viral infection [25].

The recovery of olfactory function following virus-induced damage remains poorly understood, though neural plasticity—particularly in the piriform cortex—is increasingly recognized as a key factor. Olfactory training has shown promise in promoting sensory neuron regeneration and synaptic reorganization. Neuroimaging studies, including work by Kollndorfer et al., demonstrate that such training can shift brain connectivity from non-olfactory to olfactory-specific networks, highlighting the olfactory system’s capacity for structural and functional adaptation [26]. These findings suggest that both peripheral and central mechanisms contribute to recovery, offering insights for future therapeutic strategies and biomarker development.

There are practical implications of this review for clinicians seeing patients with post-COVID-19 post-viral smell dysfunction. The evidence presented in this pooled analysis suggests that olfactory training should be recognized as a central treatment for individuals with persistent loss of smell. In addition, pairing olfactory training with anti-inflammatory agents (e.g., PEA and luteolin) may offer even better recovery outcomes, indicating that developing an individualized multimodal approach offers greater potential for improvement than olfactory training alone. Clinicians should be careful about considering pharmacological treatments such as corticosteroids because of their unpredictable efficacy and potentially more concerning undesirable effects on general health. Corticosteroids may confer benefits; however, they should only be utilized after other interventions have failed, or if they are indicated for other reasons, such as addressing inflammation that may have contributed to olfactory dysfunction [27].

The findings from this systematic review provide exciting opportunities for increased investigation; however, there are clearly limitations that must be considered as well. The included studies varied both in methodological rigor and overall quality, specifically in terms of whether blinding and randomization were applied adequately. Moreover, there were small sample sizes in some of the studies that would limit a more generalized understanding of the findings. The lack of a uniform degree of heterogeneity in some of the meta-analyses (e.g., I^2^ = 87%) indicates differences in applications of the different interventions and OT protocols, while underscoring the need for larger studies that adhere to standardized protocols. Additionally, one of the analyzed studies [23] utilized a scale that did not define a threshold for olfactory dysfunction severity, which contributes to increased heterogeneity in the reported outcomes. Likewise, follow-up periods varied across studies, and some studies only assessed short-term outcomes. Long-term follow-up studies are necessary to determine whether the use of olfactory training or paired adjunct therapies will yield enduring benefits, and to determine optimal protocols for olfactory training and the most efficacious adjunct therapies in patients with post-COVID-19-related olfactory dysfunction. Larger RCTs with standard interventions and long periods of follow-up will hopefully provide greater certainty with respect to sustained effects. Also, understanding the reasons for improvements in olfactory function, especially with combinations of therapies, may elucidate some of the relevant issues associated with the neural plasticity of smell recovery. Finally, head-to-head RCTs comparing standard therapies with emerging approaches, such as platelet-rich plasma and online olfactory training, are warranted to determine relative efficacy.

## 5. Conclusions

Olfactory training remains a promising non-pharmacological intervention for post-COVID-19 olfactory dysfunction, with significant improvements observed when combined with adjunct therapies like PEA and luteolin. However, pharmacological treatments such as corticosteroids and ALA show limited benefits, warranting further investigation. This review provides strong evidence to support the use of olfactory training in clinical practice and highlights the importance of future research to optimize treatment protocols and investigate additional adjunct therapies.

## Data Availability

Data are available from the corresponding authors upon reasonable request.

## Supplementary Materials

The following supporting information can be downloaded at https://www.mdpi.com/article/10.3390/jcm14186578/s1: Table S1: summary of the included studies; Table S2: quality assessment of the included studies.
