# Supplementary material for: Olfactory Training for Post-COVID-19 Olfactory Dysfunction: A Meta-Analysis of Efficacy and Combination Therapies
Source: J Clin Med. 2025 Sep 18;14(18):6578. doi: 10.3390/jcm14186578 (PMC12470729; doi:10.3390/jcm14186578)
Supplement: Supplementary file 1 [file jcm-14-06578-s001.zip › jcm-3830814-supplementary.pdf]

Author	Year	Country	Intervention Group N	Control Group N	Age (Mean ± SD)	Sex (M/F Ratio)	Olfactory Training Method	Training Frequency	Training Duration (weeks)	Intervention	Control	Baseline Olfactory Score (Mean ± SD, Scale)	Post-Intervention Score (Mean ± SD, Scale)	Recovered Patients (N, %)	Follow-up Duration (weeks/months)	Adverse Effects
Abdelrahman Ahmed Abdelalim et al.	2021	Egypt	50	50	29.0 (IQR 21.75–38.0)	46/54	Sniffing rose, lemon, and clove	Twice a day	3	Steroid + training	Olfactory training alone	2.0 (0.5–5.0) (both control and intervention) 2.50 (3.43)	10.0 (9.0–10.0) (intervention - 9.67 (0.76)), 10.0 (5.0–10.0) (control - 8.33 (3.82))	31 in intervention 26 in control	3 weeks	None reported
Arianna Di Stadio et al.	2022	Italy	130	55	40.37±8.58 (Intervention), 41.25±7.23 (Control)	64/121	Sniffin' Sticks with four essences (Lemon, Rose, Eucalyptus, Cloves)	Three times daily	12	Ultramicronized palmitoylethanolamide (PEA) 700 mg + luteolin 70 mg (PEA-LUT) taken orally daily + training	Olfactory training alone	20.6 ± 7.9 (intervention), 18.3 + 7.9 (control)	29.8 ± 7.5 (Intervention), 19.5 ± 7.3 (Control)	120 (92%) in Intervention, 23 (42%) in Control	12 weeks	None reported
Lorena Pinheiro Figueiredo et al.	2023	Brazil	49	51	40.37±8.58 (Intervention), 41.25±7.23 (Control)	20.4% M / 79.6% F (Intervention), 15.7% M / 84.3% F (Control)	Olfactory training with four odorants (Rose, Eucalyptus, Lemon, Cloves)	Twice daily	12	Alpha-lipoic acid (ALA) 600 mg/day combined with olfactory training for 12 weeks	Olfactory training alone	2.7 ± 1.5 (Intervention), 2.9 ± 1.4 (Control)	4.6 ± 1.3 (Intervention), 4.3 ± 1.6 (Control)	8 (16.3%) Intervention, 8 (15.7%) Control	12 weeks	Mild stomach burning and heartburn (26.5% Intervention, 7.8% Control)
Mohamed H. Abdelazim et al.	2023	Egypt	25	25	40.37±8.58 (Intervention), 41.25±7.23 (Control)	31/19 (M/F)	Olfactory training with standard odorants (Phenyl ethyl alcohol, Eucalyptol, Citronellal, Eugenol)	Twice daily (Intervention), Thrice daily (Control)	12	1% intranasal ethylene diamine tetraacetic acid (EDTA) spray combined with olfactory training	Olfactory training alone	13.76 ± 0.36 (Intervention), 13.54 ± 0.35 (Control)	18.95 ± 3.58 (Intervention), 21.06 ± 3.05 (Control)	88% (Intervention), 60% (Control)	3 months	None reported (Intervention), Mild burning and throat discomfort (Control)
Arianna Di Stadio et al.	2023	Italy	50	50	40.9 ± 11.7 (Olfactory training + placebo), 42.7 ± 13.5 (um-PEA–LUT +	26 women, 12 men (Olfactory training + placebo)	Olfactory training (Lemon, Rose, Eucalyptus, Clove)	Three times a day	12	Once-daily ultramicronized palmitoylethanolamide (PEA) 700 mg + luteolin 70 mg + olfactory training	Olfactory training alone	6.5 ± 3.7 (control), 8.2 ± 2.5 (intervention PEA–LUT + OT)	9 ± 3.6 (control), 12.8 ± 1.9 (intervention)	50 in intervention 14 in control	3 months	None reported

					olfactory training)											
Arianna Di Stadio et al.	2023	Italy	94	36	36.7 ± 11.8 (Intervention), 50.5 ± 12.7 (Control)	49 women, 45 men (Intervention), 21 women, 15 men (Control)	Olfactory training (Lemon, Rose, Eucalyptus, Clove)	Three times a day	12	Ultramicronized palmitoylethanolamide (PEA) 700 mg + luteolin 70 mg (umPEA-LUT) + training	Olfactory training alone	8.2 ± 2.7 (Intervention), 9.6 ± 2.4 (Control)	11.1 ± 2.2 (intervention), 10.1 ± 2.3 (control)	52.1% (Intervention), 18.2% (Control)	3 months	None reported
Elena Cantone et al.	2024	Italy	17	68	44.8 ± 12.2 (umPEALUT + OT), 52.1 ± 11.8 (Control)	11 women, 6 men (umPEALUT + OT), 13 women, 10 men (Control)	Olfactory training (Lemon, Rose, Eucalyptus, Clove)	Three times a day	12	umPEA-LUT + olfactory training (OT)	Olfactory training alone	18.6 ± 10.4 (umPEALUT + OT), 26.9 ± 5.3 (Control)	29.7 ± 7.5 (umPEALUT + OT), 27.7±5 (control)	96% for combination, 65% for control, 53% for umPEALUT and 29% for ALA	6 months	None reported
L. D'Ascanio et al.	2021	Italy	7	5	42.2 ± 14.1 (Intervention), not reported (Control)	5 women, 2 men (Intervention), 3 women, 2 men (Control)	Olfactory training (Sniffin' Sticks)	Twice daily	4	Ultramicronized palmitoylethanolamide (PEA) 700 mg + luteolin 70 mg + olfactory training	Olfactory training alone	21.1 ± 5.5 (Intervention), 28.8 ± 1.2 (Control)	25.2 ± 5.9 (Intervention), 31.1 ± 5.5 (Control)	2-fold improvement in the intervention group no percentage was found	1 month	None reported

OT – Olfactory Training, PEA – Palmitoylethanolamide, LUT – Luteolin, PEA–LUT / umPEA–LUT – Ultramicronized Palmitoylethanolamide + Luteolin, ALA – Alpha-Lipoic Acid, EDTA – Ethylenediaminetetraacetic Acid, SD – Standard Deviation, IQR – Interquartile Range

Table S1. Summary of the included studies

Table S2. Quality assessments of the included studies

✓, low risk of bias; ✗, high risk of bias; ~, and moderate risk of bias/some concerns

JBI Critical Appraisal Checklist For Randomised Controlled Trials													
Study	Was true randomization used for assignment of participants to treatment?	Was allocation to treatment groups concealed?	Were treatment groups similar at the baseline?	Were participants blind to treatment assignment?	Were those delivering treatment blind to treatment assignment?	Were outcomes assessors blind to treatment assignment?	Were treatment groups treated identically other than the intervention of interest?	Was follow-up complete, and if not, were differences between groups in follow-up adequately described and analyzed?	Were participants analyzed in the groups to which they were randomized?	Were outcomes measured in the same way for treatment groups?	Were outcomes measured in a reliable way?	Was appropriate statistical analysis used?	Was the trial design appropriate, and were any deviations from the standard RCT design accounted for in the conduct and analysis of the trial?
Abdelrahman Ahmed Abdelalim et al.	✓	~	✓	✗	~	~	✓	✓	✓	✓	✓	✓	✓
Arianna Di Stadio et al.	✓	✓	✓	✓	✓	✓	✓	✓	✓	✓	✓	✓	✓
Lorena Pinheiro Figueiredo et al.	✓	✓	✓	✓	✓	✓	✓	✓	✓	✓	✓	✓	✓
Mohamed H. Abdelazim et al.	✓	~	✓	✗	~	✓	✓	✓	✓	✓	✓	✓	✓
Arianna Di Stadio et al.	✓	~	✗	✓	✓	✓	✓	✓	✓	✓	✓	✓	✓
Arianna Di Stadio et al.	✓	~	✓	✓	✓	✓	✓	✓	✓	✓	✓	✓	✓
Elena Cantone et al.	✓	✓	✓	✗	✓	✓	✓	✗	✓	✓	✓	✓	✓
L. D'Ascanio et al.	✓	~	✗	✗	✗	✓	✓	✓	✓	✓	✓	✓	✓
